# Assessing the Health Effects of Long-Term Exposure to Insecticide-Treated Mosquito Nets in the Control of Malaria in Endemic Regions

**DOI:** 10.1100/tsw.2004.195

**Published:** 2004-11-19

**Authors:** Ebere C. Anyanwu, John E. Ehiri, Ijeoma Kanu, Mohammed Morad, Soren Ventegodt, Joav Merrick

**Affiliations:** Neurosciences Research, Cahers Inc., 8787 Shenandoah Park Drive, Suite 122, Conroe, Houston, TX 77385, USA; ^2^ Department of Maternal and Child Health, School of Public Health, University of Alabama at Birmingham (UAB), Birmingham, AL, USA; ^3^ 3Department of Microbiology, Abia State University, Uture, Abia State, Nigeria; ^4^ Clalit Health Services and Division of Community Health, Department of Family Medicine, Ben Gurion University, Beer-Sheva, Israel; ^5^ The Quality of Life Research Center, Teglgårdstræde 4-8, DK-1452 Copenhagen K, Denmark and The Scandinavian Foundation for Holistic Medicine, Sandvika, Norway; ^6^ National Institute of Child Health and Human Development, Office of the Medical Director, Division for Mental Retardation, Ministry of Social Affairs, Jerusalem and Zusman Child Development Center, Division of Pediatrics and Community Health, Ben Gurion University, Beer-Sheva, Israel

**Keywords:** insecticide-impregnated bednets, insecticides, malaria control, long-term exposure, health hazards, exposure, public health

## Abstract

Malaria is a protozoan disease caused in humans by the genus Plasmodium of which four species are known: *P. falciparum*, *P. vivax*, *P. ovale*, and *P. malariae*. It is transmitted through the bite of infected female mosquitoes of the genus Anopheles. Malaria is endemic in tropical and subtropical regions of the world. It is characterized by extreme exhaustion associated with paroxysms of high fever, sweating, shaking chills, and anemia. Approximately 40% of the world's population, mostly those living in the poorest nations, are at risk. Much of the deaths due to malaria occur in Africa, mostly among children. The search for prevention and control interventions that are effective and sustainable remains an abiding challenge for national governments and international health agencies. To this end, the World Health Organization and several nongovernmental organizations are investing in the use of insecticide-treated mosquito nets (ITMNs) as a viable option. Trials of ITMNs in the 1980s and 1990s showed that they reduce deaths in young children by an average of 20% and multilateral agencies, spearheaded by Roll Back Malaria (RBM), seek to have 60% of the populations at risk sleeping under ITMNs by 2005. All pesticides are toxic by nature and present risks of adverse effects that depend on toxicity of the chemical and the degree of exposure. While there is agreement that ITMNs can be effective in reducing malaria morbidity and mortality under field trials, a number of factors relating to their sustainability and contribution to health improvement in less-developed countries have yet to be determined. In particular, the adverse effects associated with their long-term use and misuse has yet to be fully evaluated. Although this paper examines potential neurotoxic and neurobehavioral effects of long-term use of ITMNs and discusses priority public health actions for protecting the health of users, it forms the basis for further research.

## INTRODUCTION

Malaria imposes a heavy burden on the health and economic productivity of populations in endemic regions (see Fig. 1). In the tropical and subtropical regions of the world, malaria causes 300–500 million clinical cases and about 2 million deaths each year. More than 90% of the deaths occur in sub-Saharan Africa and mostly among children under 5 years of age[1]. Malaria is a leading cause of perinatal mortality, low birth weight, and maternal anemia[2], while also having an economic drain both in terms of lost human productivity and healthcare costs. It is estimated that in sub-Saharan Africa, 15% of all disability-adjusted life-years (DALYs) are lost to malaria. It accounts for 40% of public health expenditure, 30–50% of inpatient admissions, and up to 50% of outpatient visits in areas with high transmission[1].

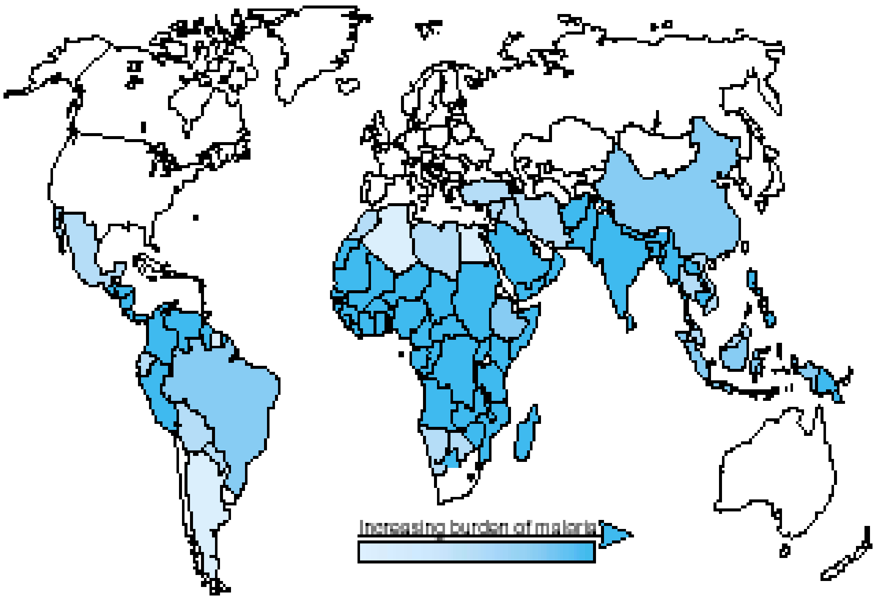

**FIGURE 1.** Malaria endemic regions. Source: Roll Back Malaria, 2002.

In addition, malaria impedes children's schooling and social development through absenteeism and permanent neurological damage associated with severe forms of the disease. The burden of malaria, combined with HIV/AIDS and tuberculosis, presents a serious challenge not only to public health in sub-Saharan Africa, but also to economic development. It has been estimated that malaria has slowed economic growth in African countries by 1.3% per year, which over 35 years amounts to a 32% reduction in the gross domestic product (GDP) of countries in the continent or an estimate at US$12 billion per year[1]. Over the last 2 decades, morbidity and mortality from malaria have been increasing due to deteriorating health systems, the impact of HIV/AIDS/TB, civil unrest, growing drug resistance, human migration, and population displacements. Thus, development of effective sustainable prevention, control strategies, and infrastructures is important.

## EMERGING STRATEGIC APPROACH

In response to the challenge posed by malaria in less-developed countries, Roll Back Malaria (RBM), a global partnership for prevention and control of malaria, was founded in 1998 by the World Health Organization (WHO), the United Nations Development Program (UNDP), the United Nations Children's Fund (UNICEF), and the World Bank. The goal was to reduce the world's malaria burden by half by 2010 with the period 2001–2010 tagged as the "*United Nations Decade to Roll Back Malaria*" (http://www.who.int/inf-fs/en/InformationSheet02.pdf). The RBM partnerships include national governments, civil society and nongovernmental organizations, research institutions, international development agencies, and development banks. One key strategy that has emerged as a tool for malaria control under the leadership of RBM and its allies is the use of insecticide-treated mosquito nets (ITMNs). As the WHO asserts, "there is no doubt about the effectiveness of ITMNs; the challenge is to scale up their use"[2]. This is based on field trials on the effectiveness of ITMNs[3,4,5,6], which demonstrated significant reductions in mortality following their use.

Preventing the death of children from malaria is so important that it would seem that any measure to achieve this deserves unequivocal commendation. However, often in international health, resources and efforts are committed and abandoned because of unanticipated problems with sustainability[7] and limited operational research suggests that this may well be case with ITMNs[8]. One area of the current initiative to promote the use of ITMNs that has received limited attention is operational research to examine the potential hazards and risks of long-term exposure to ITMNs. This is a serious omission given that products for impregnating mosquito nets with insecticides will be handled by untrained persons in uncontrolled settings in less-developed countries. This paper is an attempt to contribute to the existing body of knowledge on this subject, with the hope that the ensuing argument will provide further impetus for debate and re-evaluation of the sustainability of ITMNs as a lead tool for malaria control in sub-Saharan Africa.

## INSECTICIDES IN THE CONTROL OF MALARIA

The word insecticide is used to describe any chemical that is applied against undesired insects that cause harm to animals, humans, and plants. All pesticides are toxic in nature. The risk of adverse effects from a pesticide depends on the toxicity of the chemical and the degree to which an organism is exposed. Health risks arising from the use of ITMNs primarily include exposure during storage and transport, during net treatment, and during use; exposure to vapors; or dermal or oral contact. There is limited literature on the health hazards associated with long-term exposure to ITMNs[9,10], however, the neurotoxicity of many insecticides have been studied and reviewed in humans[11,12,13,14]. Evidence showed that all organochlorines, organophosphates, carbamates, pyrethroids, and some fumigants used as insecticides are known neurotoxicants.

The use of chemical insecticides such as organochlorines to combat mosquitoes began in the 1940s. Over the years, several research findings demonstrated that the use of chemical insecticides in controlling malaria vector had not only failed in its objective, but also induced toxic effects on humans and the environment[15]. It is unfortunate, therefore, that in less-developed countries, particularly Africa, insecticides such as DDT, Aldrin, and Diedrin are still being used without effective restriction[16]. Hence, the indoor and outdoor environmental dispersion of insecticides has become relatively ubiquitous pollutants in human tissues, drinking water, soils, and food[17]. Their adverse toxic effects are mainly due to persistence in the environmental media over time and their consequent interaction with human biological systems, which may in turn lead to a plethora of neuropathological and behavioral health consequences[18].

## USE OF INSECTICIDES IN LESS-DEVELOPED COUNTRIES

In less-developed countries, particularly sub-Sahara Africa, pesticide user health and safety procedures are almost nonexistent and never considered a serious issue in political and health discourse. During the rainy season, when the rate of malaria infection is high, people in the endemic areas use insecticides, including the “outlawed” DDT, to combat *Anopheles* mosquitoes indoors with no awareness of the health risks. Their risk perceptions are very low and self-protective behaviors are built on ignorance and limited health hazard awareness. People apply pesticides without protective masks and they usually spray or light up the insecticide “coils” in their bedrooms before going to sleep[19,20].

In a situation such as this, it would be impossible to avoid direct pesticide inhalation and at least residual neurotoxic response to the chemical insecticide. To investigate the extent of the problem, the U.K. Pesticide Action Network (http://www.pan-uk.org/default.htm) undertook case study evaluations of safe use of ITMNs in a number of African countries. In Gambia, for example, the group selected permethrin, the insecticide used to treat bednets in the country’s malaria control program[20]. Permethrin is imported into Gambia by the government and a number of aid agencies. A total of 1,300 villages are involved in the program and studies have shown that the use of permethrin-impregnated bednets has reduced the number of fatalities from cerebral malaria. However, permethrin is a neuro-poison and as well as acting on the nervous system, can cause itching and burning sensations on exposed human skin. Field studies revealed significant risks of exposure at a number of stages of the chemicals life cycle. These included when the pesticide is decanted from the large imported drums, when the bednets are impregnated by village health workers or by individuals in their own homes, when unlabelled containers are brought into the home, and from misuse of the chemical[20].

### Pyrethrins: Permethrin

Because of the adverse toxic effect of chemical insecticides, it became necessary to look for suitable compounds that would be efficacious, environmentally friendly, and nontoxic to humans. Unfortunately, “no such suitable alternative exists”[21]. Consequently, chemists resorted to such natural plant products as “alternative insecticides”; hence, pyrethrins obtained largely from the flowers of *Chrysanthemum cinerariaefolium* plant were found to be effective[22]. The insecticidal activity of pyrethrin flowers has been known for over 2,000 years, but the commercial production of pyrethrum began about 2 centuries ago[21,23]. However, it was only in the 1920s that the chemical structure of the active insecticidal ingredient was demonstrated in the plant [23]. demonstrated the chemical structure of the active insecticidal ingredient in the plant. Kenya is the world’s largest producer of *C. cinerariaefolium*. Other major producers are Russia and Japan. Each flower head contains 2–4 mg (c. 2%) of a mixture of active components that are extracted with light petroleum. It is from the chlorination of *C. cinerariaefolium* that permethrin used in insecticide-impregnated bednets is derived[21,23].

### Pyrethrins Analogs

Several research activities were carried in the 1970s to synthesize analogs of pyrethrins, but it was Elliot and Janes[24] at the Rothamsted Research Station that made a breakthrough. Several compounds of pyrethrins were prepared including allethrin (prepared from a racemic mixture of (R, S)-chrysanthemic acid) and bioresmethrin (in which the cyclopentenone ring of the natural product was replaced by the structurally simpler benzylfuran and yet the compound was extremely potent, although still short-lived because of its photosensitivity)[24]. The success of Elliot and Janes[24] led to the commercial development of permethrin[26]. Permethrin is supplied and marketed as an insecticide under several trade names, for example, Ambush (ICI), Cooper powder (Wellcome Foundation Ltd), Permit (Pan Britannica Ltd), Rentokil Musk control, and in mixtures with other substances[23].

### Permethrin-Impregnated Bednets

Field trials of permethrin-impregnated bednets against malaria vectors began about 2 decades ago and several follow-up studies have been conducted and reported[27,28,29,30,31]. While results indicate reductions in mortality in the short term, little research has been conducted on the biomedical hazards associated with use of ITMNs and the long-term health effects of their use. Research attention has been meager and data on acute and chronic health effects related to their toxic exposures are generally lacking[32], apart from a few recent reviews[9,33,34]. This suggests a need for a major research focus, with priority on long-term effects, particularly cancer, neurodevelopmental and neurobehavioral effects, long-term neurological dysfunction, and reproductive outcomes. No studies have been done or reported specifically on the clinical manifestations of ITMN neurotoxicity and neurobehavioral disorders at lower exposure concentrations. Suitable populations at high risk should be studied, including noncertified pesticide applicators, seasonal and permanent casual users, and their children. It is of interest to public health especially that the users of insecticide-impregnated bednets be made aware that long-term exposure to an environmentally active neurotoxicant can be hazardous to health.

## NEUROTOXICITY AND NEUROBEHAVIORAL EFFECTS

All insecticides have both acute and chronic effects[35] and their use in agriculture and industry is largely regulated[36]. However, in spite of the known health risks associated with these, our review of reports on the benefits of ITMNs did not yield any information relating to the potential adverse effects of long-term exposure. A given public health intervention may have proven effectiveness in reducing morbidity and mortality, but only carefully planned studies examining negative impacts can reveal long-term adverse effects. The promotion of ITMNs as an important component of a malaria control program in malaria-endemic regions of the world should include studies on effects of long-term exposure. In the absence of such data, there would seem to be little justification for their continued use as a sustainable method for controlling malaria infection. The human central nervous system (CNS) and the peripheral nervous system (PNS) comprise highly specialized organs and tissues, which are extremely sensitive to all chemical pollutants and can be injured or impaired by insecticide inhalation[37]. Certain features of neural tissues make them particularly susceptible to insecticide effects: they are unable to replace lost and damaged cells; some of their components, such as myelin, have the tendency to accumulate and retain lipophilic substances, such as organic solvents; and, they have a large surface area, which increases the likelihood of exposure to systematically distributed neurotoxicant[38]. Potentially serious neurotoxic effects of insecticides include peripheral neuropathy, extrapyramidal disorders, ataxia, sensory disorders, psychosis, CNS depression, and in extreme cases, death[39]. The physiological responses might not be manifest as overt disease effects, however, subclinical behavioral or neurological changes can be early indicators of an important disease process and can help identify persons affected by exposures at stages that offer more effective opportunities for intervention[40].

Like other naturally occurring pesticides, all pyrethroids (including permethrin) are capable of affecting the heart rate of adult humans at as little as 2 mg and can cause loss of memory[41]. Serious poisonings are frequent and in extreme cases may lead to death from respiratory failure[21,26,42]. Their toxic action takes place at the synapses, or junctions between nerve cells, where impulses are transmitted chemically through the acetylation of chlorine. When functioning normally, this reaction is rapidly reversed through the action of cholinesterase, so that stimulation ceases and the site is ready to receive a new impulse. The pyrethrins and other insecticides poison the nerves by inhibiting cholinesterase, which leads to the failure of nervous transmission, convulsions, and death[21,26,42]. Metabolically, their action on the nervous system may probably lead to hallucination, myoclonic phenomena, migrainous headaches, nausea, emesis, genetic mutation, and reproductive dysfunction.

## CANCER RISKS

The potential danger of human exposure to pesticides has become increasingly clear in the last 25 years and the association with cancer types[43,44] is now evident in several cancer registers in developed countries. In Africa, however, there are scarce (if any) cancer registers and records of cancer deaths are limited. Furthermore, cancer cases are very rarely reported as opposed to the developed countries, where strict regulations are in force. Probably, permethrin may cause damage to the immune system and lead to chromosomal abnormalities. It may possibly modify the functions of liver enzymes and interfere with the conduction of nervous impulse.

Permethrin's cancer-causing capability probably depends on the length of exposure as shown hypothetically in Fig. 2. This may occur when hydrogen atoms in the nucleotide bases undergo slight alterations in position. Hypothetically, for example, permethrin may act on the chlorine atoms of nucleic acids thereby causing mutation. In addition, this nucleic complex may act as immunosuppresant[45]. On the other hand, permethrin may displace adenine and if this replacement takes place during reproduction, mutation of the genes may occur (schematically presented in Fig. 2). Note that the CG base pair (II) replaces the original AT (III, IV) base pair at the top of the DNA molecule, probably due to interference of permethrin. The DNA molecule replicates in the normal fashion, except that the permethrin that replaced the A mispairs with G, rather than with T, as A normally does. When this happens, the base is unable to pair with its normal complementary partner, but instead pairs with another base; for example, guanine may pair with thymine, or cytosine with adenine. If permethrin is incorporated in place of adenine, it may pair with guanine instead of thymine, causing a base substitution during cell division.

Normally, tautomeric shifts are only transitory states of a base and have little effect. If, however, a tautomeric shift occurs at the time of DNA replication, the mispairing causes a change in the base sequence in the complementary chain that can be transmitted to future copies of the DNA — a substitution mutation. Tautomeric shifts occur because of natural chemical properties of the nucleotide bases and help explain the normal spontaneous mutation rate. Like many other environmental agents, all insecticides also affect the rate of mutation. Additionally, variations in the mutation rate have been associated with biological factors such as age and sex[46]. Under these circumstances, it is possible that unidentified genetic diseases may occur in mating involving older fathers. The people who live in malaria-endemic regions of Africa are already environmentally exposed to insecticides and the additional exposure to ITMNs could enhance a tissue saturation point and increase the rate of genetic mutation since all chlorinated hydrocarbons are powerful mutagens[47].

## PUBLIC HEALTH PRIORITY ACTION

From the points raised so far, it is imperative that international health agencies and national governments re-evaluate the application of ITMNs as one of the strategies for combating malaria vectors, by instituting studies of long-term effects. One of the major problems reported in previous trials in Africa was the lack of awareness of pesticide-related health risks and hazards[48]. It is important that people are made aware that exposure to insecticides places them at increased risk of a variety of acute and chronic conditions, including cancer[49,50]. Hazard characterization involving the re-examination of all available experimental animal and human data and the associated doses, routes, timing, and duration of exposure to determine qualitatively if and which brand of insecticide causes neurobehavioral and neurotoxic effects and under what conditions must be undertaken.

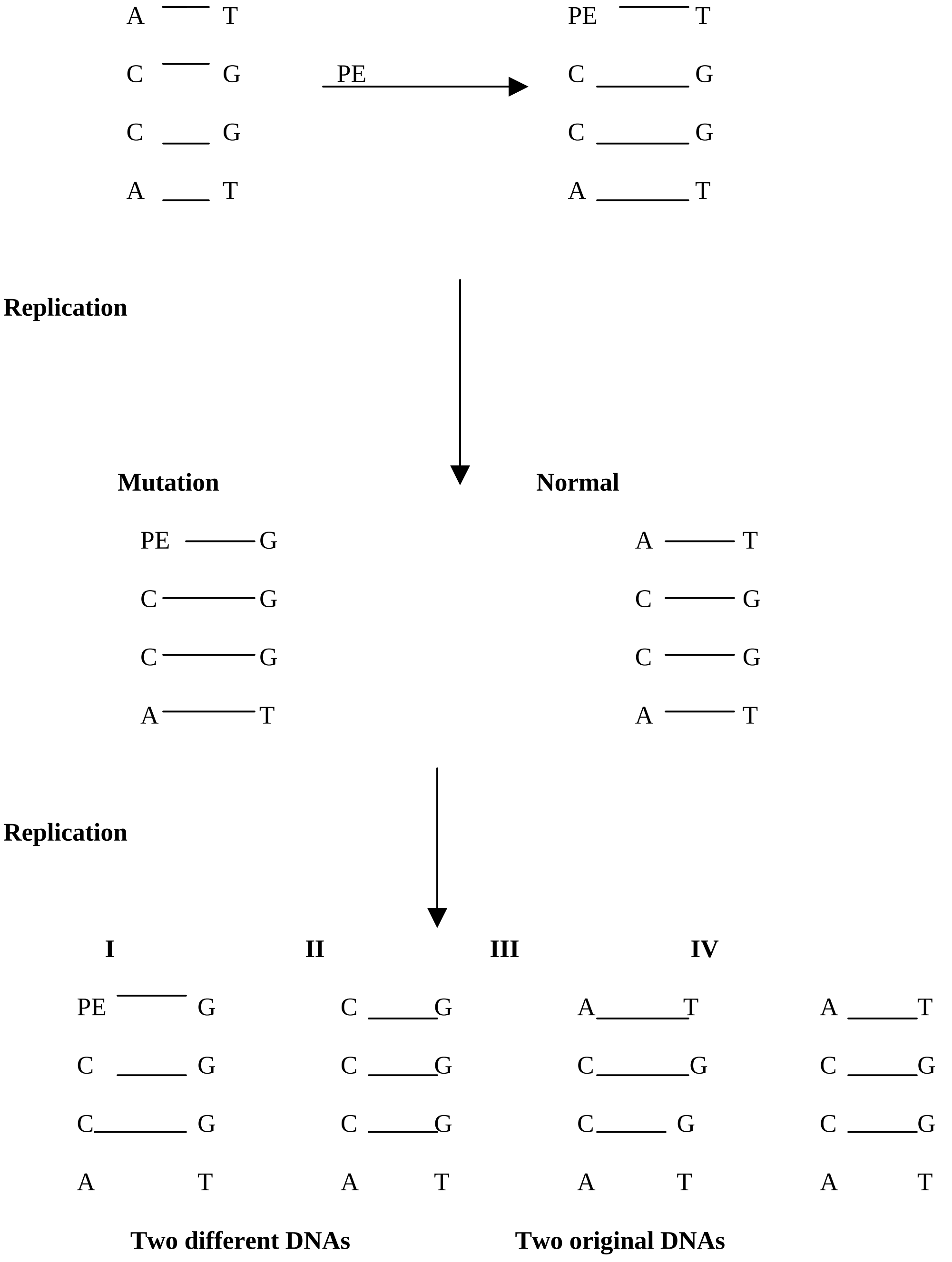

**FIGURE 2.** Hypothetical genetic mutation due to the displacement of nucleic acid in the DNA by permethrin. This process is called tautomeric shift. Observe that permethrin reacts with nucleic acid to form a permethrin-nucleic acid complex. In fact, two chlorine atoms from permethrin react with the two hydrogen atoms to form guanine and cytosine, respectively.

It is possible that, from the hazard characterization and other relevant criteria, the health-related database can be generated and characterized as sufficient or insufficient for use in risk assessment. By combining hazard identification and some aspects of dose-response evaluation into hazard characterization, the evaluation and use of data for other purposes when quantitative information for setting reference doses (RfDs) and reference concentrations (RfCs) is not available will not be precluded[3,44,45,49,50]. Exposure assessment is important since it will assist in identifying human populations exposed or potentially exposed to insecticides, describe their composition and size, and present the types, magnitudes, frequencies, and duration of exposure to insecticide hazards. The exposure assessment would provide an estimate of human exposure levels for particular populations from all potential sources.

Since there had not been any scientific judgments made concerning the potential for insecticide-impregnated bednets to cause health problems in humans, it is important that studies are carried out in this regard. Such research should adopt the National Research Council (NRC) recommendations, which defines risk assessment as including some or all the following components: hazard identification, dose-response assessment, exposure assessment, and risk characterization[21,51,52,53]. Based on these recommendations, serious and urgent evaluation of insecticide-impregnated bednets should be conducted through a research approach that is less fragmented and more holistic, and more interactive, and that deals with recurring conceptual issues that cut across all stages of risk assessment. Such research should describe a more interactive approach by organizing the process around the qualitative evaluation of toxicity data (hazard characterization), the quantitative dose-response analysis, exposure assessment, and the risk characterization. The hazard characterization should include deciding whether a pesticide has an effect by means of qualitative consideration of dose-response relationships, route, and duration of exposure. Efforts should be made to obtain information regarding personal characteristics, life style factors, and other variables that might be necessary for the risk assessment. Descriptive statistics of concentration levels should be computed to characterize the current extent of contamination. Time trends should be followed across the years and homogeneous subgroups should be compared using multiple regression analysis to investigate associations between determining factors and specific insecticides. This approach would combine the information important in comparing the toxicity of ITMNs with potential human exposure scenarios.

## CONCLUSION

Even in the interest of public health, the use of insecticides without adequate risk assessment is unacceptable. Getting 60% of the world's population at risk of malaria sleeping under ITMNs as targeted by RBM is a major operation that would involve unprecedented use of insecticides in less-developed countries. The negative effects of long-term exposure to these chemicals need to be balanced against the benefits from decreasing the burden of disease. In doing this, it has to be noted that in resource-poor settings, particularly Africa, what can be perceived as minimal risk may indeed become a major public health catastrophe when the risk combines with poverty, ignorance, illiteracy, and a chronic lack of resources for dealing with emergencies. Thus, a reassessment of the health risks associated with ITMNs is particularly important. In weighing the benefits of ITMNs against risks associated with their use, international development and health agencies should pay equal attention to other strategies that rely on environmental management and human behavior. Rather than conclude that the risks associated with long-term use of ITMNs are minimal compared with lives saved from malaria[9], a program of research should be instituted to identify the effects of long-term use on different segments of the population.

Procedures for monitoring exposure to ITMNs need to be instituted so that monitoring could be carried out on a regular basis, and to facilitate assessment of impacts and changes over time. Human behavior and the potential for unapproved misuse of insecticides should be addressed. Risk analyses should be based on actual levels of health and environmental exposure under the conditions of use. Training (including training-of-trainers) in insecticide use and management is important at all levels, and methods of disseminating information and knowledge about insecticides need to be instituted. Public awareness of potential risks associated with use and misuse of insecticides, and of the health impacts insecticides is essential in developing countries as it is in industrialized countries[3,21,34]. Given the potential risks associated with insecticide programs, promotion of ITMNs ought be subject to health impact assessment (HIA).

## BIOSKETCHES

**Ebere C. Anyanwu, PhD** is a research scientist in the Medical Center for Immune and Toxic Disorders and also adjunct Professor of Anatomy and Physiology at the North Harris and Montgomery College District, Houston, TX. E-mail: ebereanyanwu@msn.com

**John Ehiri, PhD, MPH** is an Assistant Professor in the Department of Maternal and Child Health, School of Public Health, University of Alabama at Birmingham (UAB), Birmingham, AL. E-mail: jehiri@uab.edu.

**Ijeoma Kanu, MSc., Ph.D** (Candidate) is a lecturer in Department of Microbiology, Abia State University, and a PhD Research Student in environmental microbiology at the Michael Okpara University of Agriculture, Umudike, Umuahia, Abia State Nigeria. E-mail: ijay2001kay@yahoo.com

**Mohammed Morad, MD** is a specialist in family medicine, lecturer in family medicine at the National Institute of Child Health and Human Development; Division of Community Health, Ben Gurion University of the Negev and the medical director of a large area clinic in the city of Beer-Sheva. Publications include those on Bedouin health, health aspects, spiritual health and aging in persons with intellectual disability, and he is a presenter on topics like health policy and services for disadvantaged at national and international conferences. E-mail: morad62@barak-online.net

**Søren Ventegodt, MD,** is the Director of the Quality of Life Research Center in Copenhagen, Denmark. He is also responsible for a Research Clinic for Holistic Medicine in Copenhagen and is a popular speaker throughout Scandinavia. He has published numerous scientific or popular articles and a number of books on holistic medicine, quality of life, and quality of working life. His most important scientific contributions are the comprehensive SEQOL questionnaire, the very short QoL5 questionnaire, the integrated QOL theory, the holistic process theory, the life mission theory, and the Danish Quality of Life Research Survey, 1991–94 in cooperation with the University Hospital of Copenhagen and the late pediatric professor Bengt Zachau-Christiansen. E-mail: ventegodt@livskvalitet.org. Website: www.livskvalitet.org/

**Joav Merrick, MD, DMSc,** is Professor of Child Health and Human Development affiliated with the Zusman Child Development Center, Division of Pediatrics and Community Health at the Ben Gurion University, Beer-Sheva, Israel; the Medical Director of the Division for Mental Retardation, Ministry of Social Affairs, Jerusalem; and the Founder and Director of the National Institute of Child Health and Human Development. He has numerous publications in the field of child and human development, rehabilitation, intellectual disability, disability, health, welfare, abuse, advocacy, quality of life, and prevention. Dr. Merrick received the Peter Sabroe Child Award for outstanding work on behalf of Danish Children in 1985 and the International LEGO-Prize ("The Children's Nobel Prize") for an extraordinary contribution towards improvement in child welfare and well being in 1987. E-mail: jmerrick@internet-zahav.net. Website: www.nichd-israel.com
